# Enhancement in Phospholipase D Activity as a New Proposed Molecular Mechanism of Haloperidol-Induced Neurotoxicity

**DOI:** 10.3390/ijms21239265

**Published:** 2020-12-04

**Authors:** Marek Krzystanek, Ewa Krzystanek, Katarzyna Skałacka, Artur Pałasz

**Affiliations:** 1 Department and Clinic of Psychiatric Rehabilitation, Department of Psychiatry and Psychotherapy, Faculty of Medical Sciences, Medical School of Silesia in Katowice, Ziołowa 45/47, 40-635 Katowice, Poland; 2 Department of Neurology, Faculty of Medical Sciences, Medical School of Silesia in Katowice, Medyków 14, 40-772 Katowice, Poland; ekrzystanek@sum.edu.pl; 3 Institute of Psychology, University of Opole, Kopernika 11A Street, 45-040 Opole, Poland; katarzyna.skalacka@uni.opole.pl; 4 Department of Histology, Faculty of Medical Sciences, Medical School of Silesia in Katowice, Medyków 18, 40-752 Katowice, Poland; artiassone@gmail.com

**Keywords:** phospholipase D, haloperidol, chlorpromazine, fluphenazine, neurotoxicity, olanzapine, neuroprotection

## Abstract

Membrane phospholipase D (PLD) is associated with numerous neuronal functions, such as axonal growth, synaptogenesis, formation of secretory vesicles, neurodegeneration, and apoptosis. PLD acts mainly on phosphatidylcholine, from which phosphatidic acid (PA) and choline are formed. In turn, PA is a key element of the PLD-dependent secondary messenger system. Changes in PLD activity are associated with the mechanism of action of olanzapine, an atypical antipsychotic. The aim of the present study was to assess the effect of short-term administration of the first-generation antipsychotic drugs haloperidol, chlorpromazine, and fluphenazine on membrane PLD activity in the rat brain. Animals were sacrificed for a time equal to the half-life of the antipsychotic drug in the brain, then the membranes in which PLD activity was determined were isolated from the tissue. The results indicate that only haloperidol in a higher dose increases the activity of phospholipase D. Such a mechanism of action of haloperidol has not been described previously. Induction of PLD activity by haloperidol may be related to its mechanism of cytotoxicity. The finding could justify the use of PLD inhibitors as protective drugs against the cytotoxicity of first-generation antipsychotic drugs like haloperidol.

## 1. Introduction

Phospholipase D (PLD) activity in carrot cells (EC 3.1.4.4.) was first described in 1947 [1]. Until the end of the 1970s, there was a belief that PLD was an enzyme found only in plants [1]. In 1979, Taki and Kanfer described the presence of olein-dependent PLD in rat brain tissue [2]. The discovery of PLD in mammals triggered an avalanche of research to determine the place of PLD in cell physiology and pathology.

It is now known that PLD is an enzyme present in all known mammalian cells except for some leukocytes [3,4]. PLD occurs in most cellular organelles: in the plasma membrane, the Golgi network, secretory vesicles, lysosomes, the nucleus, microsomes, and mitochondria. Low PLD activity was also detected in cytoplasm [5].

**Figure 1:**
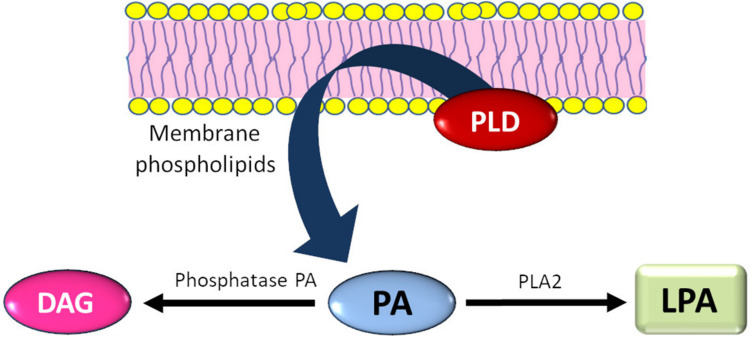
Phospholipase D (PLD)-dependent secondary messenger system. DAG = diacylglycerol, PA = phosphatidic acid, PLA2 = phospholipase A2, LPA = lysophosphatidic acid.

PLD acts on various membrane phospholipids, mainly phosphatidylcholine (PC), from which phosphatidic acid (PA) and choline are formed. Choline released by PLD may be a substrate for the production of acetylcholine in nervous and muscular tissue [6]. In turn, PA seems to be a key element of the PLD-dependent secondary messenger system (Figure 1) [7]. In animal tissues, phospholipase D occurs mostly in a form bound to cell membranes [8]. Membrane PLD has specificity for PC.

Several dozen years of research into the role that PLD plays in cell life has determined that the secondary messenger system associated with PLD is involved in a number of cellular processes. Among others, these processes include mitogenesis and cell differentiation; axon formation; synaptogenesis and myelination during CNS embryogenesis; polymerization of actin fibers and change in the shape of cells; transport of proteins from the endoplasmic reticulum to the Golgi apparatus; formation of secretory vesicles between the dictyosomes and their exocytosis; secretion of secretory vesicles in neutrophils, mast cells, and islet cells; stimulation of protein kinases (among others: N protein kinases, protein kinase C (PKC) isoforms, β-adrenergic receptor kinases, tyrosine phosphatase); regulation of small GTP activity; neurodegeneration; apoptosis; peroxide production in human neutrophils; and platelet aggregation [1,2,3,4,6].

The variety of cellular processes associated with PLD indicates its important role in systemic cytophysiology, including the central nervous system. PLD activity may also play a role in cognitive processes—It has also been observed that rats deprived of PLD were characterized with impaired social behavior and difficulty in recognizing objects [9]. After behavioral stimulation, these animals also showed a reduced acetylcholine release in the hippocampus compared with a control.

Our previous studies have shown that the long-term atypical neuroleptic olanzapine reduces PLD activity in the rat brain, which may be related to the neuroprotective effect of olanzapine [10]. Considering the differences between typical and atypical antipsychotics, we decided to check if first-generation antipsychotics act on PLD activity in a similar way to olanzapine. The present study presents the results of the single administration of chlorpromazine, fluphenazine, and haloperidol on membrane PLD activity in the rat brain.

## 2. Materials and Methods

The experiments were carried out on male Wistar rats (WIST: Mol Wu, Møllegaard, Denmark) from the in-house breeding at the Pharmacology Department of the Medical School of Silesia in Katowice-Ligota. The weight of the rats (*n* = 56) was 220 ± 10 g (s.d.). The study was approved by the Local Ethics Committee for Animal Experiments of the Silesian Medical University in Katowice (consent of 27 March 2001; No. 4/01), and we confirm that all methods used in the experiments were performed in accordance with the relevant guidelines and regulations.

The experiment consisted of short-term (one-dose) drug administration. Representative first- generation antipsychotics, pharmacologically acting mainly by blocking dopamine type 2 (D_2_) receptors, were used for the experiments. Rats received antipsychotic drugs at the following doses in terms of free compounds (the equivalent doses in mg/kg intramuscularly are given in brackets):

- chlorpromazine (CPZ) in doses of 10 mmol/kg (3.2 mg/kg) and 20 mmol/kg (6.4 mg/kg),
- fluphenazine (FLU) at 10 mmol/kg (4.35 mg) and 20 mmol/kg (8.7 mg/kg),
- haloperidol (HAL) in doses of 10 mmol/kg (3.75 mg/kg) and 20 mmol/kg (7.5 mg/kg).

In this group, FLU had the highest affinity for the D_2_ receptor (K_i_ = 0.33 nM), followed by HAL (K_i_ = 1.8 nM), and CPZ (K_i_ = 11 nM) [11]. To be able to draw reliable translational conclusions, the doses of drugs used in the experiment were the same as the doses of drugs used in the literature [12,13,14,15]. It was decided to express doses in millimoles per kilogram (mmol/kg). With this assumption, a similar number of molecules for each drug fell on the same body weight of the animal. The proposed method of drug administration made it easier to compare the effects of drugs with each other. While examining each drug, two doses were administered to differentiate the dose-dependent enzyme response. Eight rats were taken per dose, and eight as a control group with saline solution intramuscular (i.m.) administration.

Brain plasma membranes were obtained according to Jelsema [16] in the Strosznajder and Strosznajder modification [17]. Briefly, decapitation was performed on each animal after a time period equivalent to the half-life of the examined compound in the rat brain. The rationale for this was to obtain a similar distribution of the investigational drugs in the brain tissue. After decapitation, the rat brain was rapidly removed and put on ice. The dissected brain cortex was homogenized, and the homogenate was centrifuged for 3 min at 1100× *g*. The obtained supernatant was centrifuged for 10 min at 17,000× *g* to obtain a precipitate. Subsequently, the precipitate underwent hypotonic shock; then it was vortexed and centrifuged for 20 min at 48,000× *g*. The resulting pellet contained brain plasma membranes. The pellet was resuspended in a modified buffer (50 mM Tris-HCl, 1 mM MgCl_2_, 1 mM EGTA, pH = 7.2), and then immediately frozen at −70 °C for further experiments. Total protein concentration in the tested material was determined using the Lowry method. The method of determining phospholipase D activity in the plasma brain membranes of the rats was carried out according to the procedure described by Zhou et al. [18] with our own modification [19].

Briefly, PLD cleaves lecithin to yield choline and phosphatidic acid. Next, choline is oxidized by choline oxidase to betaine and H_2_O_2_. Finally, H_2_O_2_ reacts with N-acetyl-3,7-dihydroxyphenoxazine in a 1:1 stoichiometry to produce a fluorescent product, resorufin. The latter reaction is catalyzed by horseradish peroxidase. The experimentally established optimal reaction time for the determination of PLD activity was 2 min, and the optimal amount of protein in the test sample was 25 μg [19]. Fluorometric measurements were made with the Ascent FL Fluoroscan, Type 374 (Labsystems, Finland). Filters used in the experiments were set for excitation and emission at 560 and 590 nm, respectively. The standard reaction mixture (200 μL) contained 50 mM Tris-HCl (pH = 7.2), 1 mM MgCl_2_, 1 mM EGTA, 100 μM N-acetyl-3,7-dihydroxyphenoxazine, 2 U/mL HRP, 0.2 U/mL choline oxidase, 0.5 mM lecithin, and 4 mM sodium oleate. Assays were carried out in triplicate at a temperature of 37 °C (the coefficient of variation percent (CV%) = 4–7%).

In statistical calculations, the obtained results for both doses of drugs and for the control group were analyzed for multiple comparisons. The Shapiro–Wilk test of normality and Levene’s test were statistically insignificant in all analyzed groups (*p* > 0.05), so a series of one-way analysis of variance (ANOVA) with post hoc tests was made.

## 3. Results

**Figure 2:**
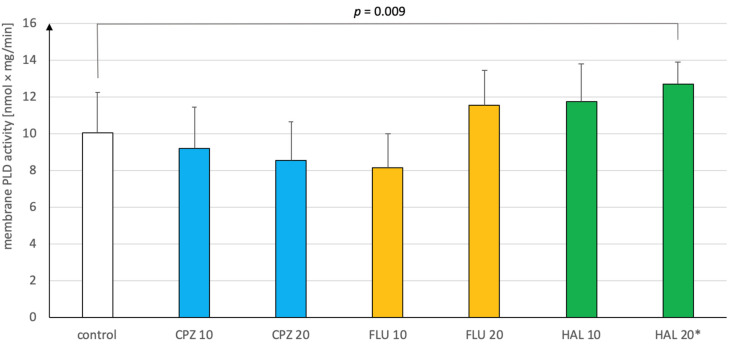
The effect of first-generation antipsychotic drugs administered in a short-term experiment on membrane phospholipase D (PLD) activity in the rat brain. Information on statistical calculations is provided in the text. CPZ = chlorpromazine, FLU = fluphenazine, HAL = haloperidol. * *p* < 0.01.

Chlorpromazine (CPZ) in our short-term experiment reduced the PLD activity in a dose-dependent manner, however, this result was not statistically significant for both doses used compared to the control group ((2,21) = 1.25; *p* = 0.31; η^2^ = 0.11). Fluphenazine (FLU) had a significant effect on the activity of the PLD tested (F(2,21) = 7.98; *p* = 0.003; η^2^ = 0.43). Post hoc Tukey tests showed that FLU at a dose of 10 mmol/kg significantly reduced the PLD activity compared with the dose of 20 mmol/kg (*p* = 0.002), but not in comparison to the control group (*p* = 0.09). Haloperidol (HAL) also had a significant effect on the activity of the PLD tested (F(2,21) = 5.48; *p*= 0.01; η^2^ = 0.34). Post hoc Tukey tests showed that HAL at a dose of 20 mmol/kg significantly increased membrane PLD activity in the rat brain in comparison with the control group (*p* = 0.009). The dose of 10 mmol/kg also had an increasing effect, but the difference was insignificant when compared to the control group (*p* = 0.11) and to the 20 mmol/kg dose (*p* = 0.48). The results are shown in Figure 2.

## 4. Discussion

A review of the literature from recent years indicated a low interest of researchers in PLD. In our opinion, further exploration of the role of PLD in the mechanisms of drug development can be important not only for development of knowledge itself, but also for practical benefits in finding new target points for new psychotropic drugs. For this reason, we decided to publish the interesting results of data from a short experiment in which we administered to rats only one dose of first-generation antipsychotic drugs.

CPZ in our short-term experiment did not significantly inhibit PLD activity. The potential involvement of CPZ in changes in PLD activity may be indicated by observations of changes in PA synthesis in human thrombocytes. They indicate inhibition of PA formation under the influence of CPZ [20]. This importance of CPZ for phospholipid metabolism, however, is contradicted by the lack of CPZ’s effect on choline turnover in human platelets, often treated approximately as a peripheral serotonin cell model [21]. Blocking of D2 receptors by CPZ may have an impact on other intracellular transmission pathways associated with phospholipid metabolism. For example, blocking of these receptors by CPZ may be responsible for reducing phospholipase C activity [22]. Perhaps CPZ administered at a higher dose than in our experiment or over a longer period may significantly reduce PLD activity. In our previous study with olanzapine in a short-term experiment, it did not significantly affect the activity of PLD. Only chronic administration for a period of 3 weeks at a dose of 10 mmol/kg resulted in a significant decrease in PLD activity [10].

There are no reports in the medical literature regarding the effect of FLU and HAL on the activity of membrane phospholipase D. In the conducted studies, FLU did not significantly affect the activity of membrane PLD.

The most interesting finding seems to be the increase in PLD activity by HAL, contrary to the non-significant decrease of PLD activity by olanzapine (OLZ) in our study and the aforementioned results of previous research with CPZ [20]. Our results mean that at higher doses, HAL may induce an intracellular transmission pathway associated with PLD. The literature is also silent about the potential consequences of such an action of HAL. Perhaps increasing the activity of secondary messengers associated with PLD by higher doses of haloperidol may contribute to neurotoxicity of HAL. The neurotoxic effects of HAL at all doses via many molecular mechanisms has been confirmed in many studies [23]. The premises behind such thinking are only indirect, as it has been observed that long-term cerebral ischemia causes increased PLD activity [24]. In this context, however, it is worth noting the potential pharmacological significance of phospholipase D inhibitors [25] like halopemid, a dual blocker PLD1/2 tested in the 1970s [26]. Selective inhibitors of individual isoforms were also sought [27]. At present, however, drugs with this mechanism of action have not entered clinical practice.

**Figure 3:**
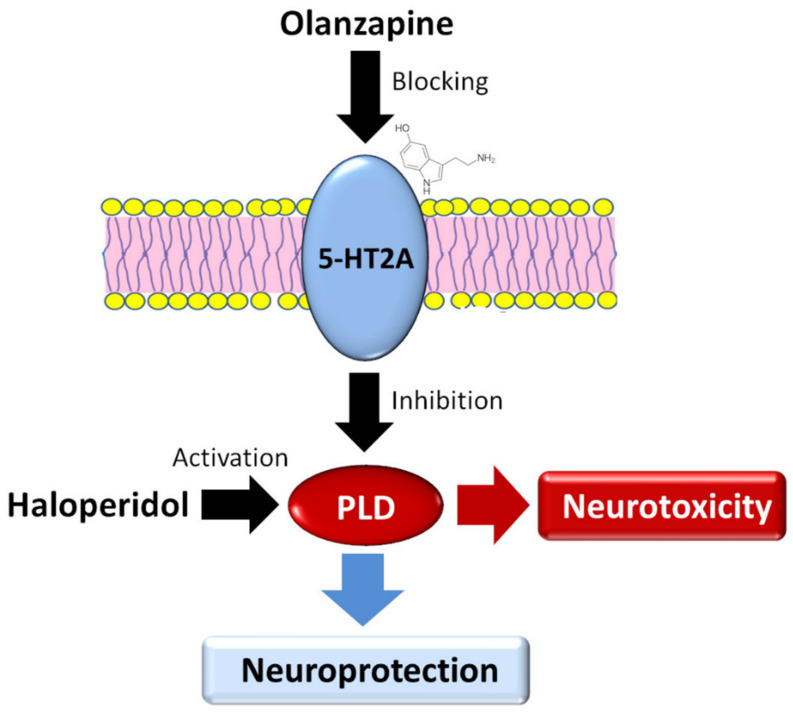
Proposition of a new molecular model of haloperidol neurotoxicity. Olanzapine, by blocking serotonin 2A (5-HT_2A_) receptors, reduces the activity of phospholipase D (PLD), and therefore has a neuroprotective effect. In turn, haloperidol, having no effect on 5-HT2A receptors, increases the activity of PLD, which may be involved in the mechanism of its cytotoxicity.

In previous studies, we showed that olanzapine reduces the activity of PLD, and the inhibition of PLD is mediated via serotonin type 2A receptors [10]. If the enhancement of PLD activity by HAL induces the neurotoxicity process, the opposite effect of olanzapine to haloperidol would support its neuroprotective properties. Research confirms the neuroprotective effect of olanzapine and other second-generation antipsychotics mediated via multiple molecular mechanisms [28]. Importantly, this mechanism is related to the blocking of serotonin type 2A receptors by second-generation antipsychotics. Inhibition of PLD activity, as opposed to the effect caused by HAL, may be another proposed mechanism of olanzapine’s neuroprotective action. Olanzapine could have a neuroprotective effect because, unlike haloperidol, it inhibits and does not increase the activity of PLD (Figure 3).

The authors are aware of the limitations of the conducted research. PLD activity was assessed in isolated plasmatic membranes, and we used a method of direct evaluation of PLD activity, which proved successful in our previous studies [10]. However, the issue of whether the inhibition of PLD activity is due to direct HLA action on the enzyme is something that needs to be confirmed by using PLD blockers or indirect methods, and by measuring PLD-specific products. Moreover, in our study, drugs were administered only once, which does not reflect the clinical situation in which patients with schizophrenia receive chronic antipsychotic medication. The results we obtained must be confirmed in a long-term study that we have not conducted. However, we believe that the demonstrated increase in phospholipase D activity by haloperidol in this short-term experiment is highly likely to indicate the possibility of the same effect with chronic administration of this drug.

## Abbreviations

PLD	phospholipase D
PC	phosphatidylcholine
PA	phosphatidic acid
CPZ	chlorpromazine
FLU	fluphenazine
HAL	haloperidol
